# Geographical Variations in Early Onset Colorectal Cancer in the United States between 2001 and 2020

**DOI:** 10.3390/cancers16091765

**Published:** 2024-05-01

**Authors:** Yazan Abboud, Madison Fraser, Imran Qureshi, Shivani Srivastava, Ibrahim Abboud, Benjamin Richter, Fouad Jaber, Saqr Alsakarneh, Ahmed Al-Khazraji, Kaveh Hajifathalian

**Affiliations:** 1 Department of Internal Medicine, Rutgers New Jersey Medical School, Newark, NJ 07103, USA; mf1124@njms.rutgers.edu (M.F.); iaq5@njms.rutgers.edu (I.Q.); ss4413@njms.rutgers.edu (S.S.); 2 School of Medicine, University of California Riverside, Riverside, CA 92521, USA; brhoum.abboud@gmail.com; 3 Division of Gastroenterology and Hepatology, Rutgers New Jersey Medical School, Newark, NJ 07103, USA; bir23@njms.rutgers.edu (B.R.); aa2758@njms.rutgers.edu (A.A.-K.); kh852@njms.rutgers.edu (K.H.); 4 Department of Internal Medicine, University of Missouri-Kansas City, Kansas City, MO 64110, USA; fouad.jaber@umkc.edu (F.J.); s.alsakarneh@umkc.edu (S.A.)

**Keywords:** colorectal cancer, early-onset colorectal cancer, geography, incidence, disparities, epidemiology, adenocarcinoma, neuroendocrine tumors

## Abstract

**Simple Summary:**

Colorectal cancer is one of the leading causes of cancer-related deaths in the US. Lately, there has been a rise in colorectal cancer in younger patients; however, there is a paucity of data on geographical variations of early-onset colorectal cancer in the US. Thus, our study aimed to evaluate the temporal change in early-onset colorectal cancer incidence rates in different regions in the US and assess these trends by sex and histopathological subtypes. We analyzed data from 2001 to 2020 from the United States Cancer Statistics database, which encompasses nearly 98% of the US population. Our results demonstrated that early-onset colorectal cancer incidence rates and time trends increased in men and women across different regions in the US, with the steepest increase noted in the west and the least in the south. These findings persisted across both main colorectal cancer histopathological subtypes (adenocarcinoma and neuroendocrine tumors) with neuroendocrine tumors showing a more pronounced increase compared to adenocarcinoma, especially in the west and northeast. Our findings hold public health implications prompting healthcare policies and future research to investigate any disproportional exposure to region-specific risk factors over the past two decades in the US, especially in western regions.

**Abstract:**

Background: Colorectal cancer remains the second leading cause of cancer-related death in the US. As early-onset colorectal cancer (EO-CRC) becomes more prevalent in the US, research attention has shifted towards identifying at-risk populations. Previous studies have highlighted the rising rate of early-onset adenocarcinoma (ADC) and neuroendocrine tumors (NET) in the US. However, data on geographical variations of EO-CRC are scarce. Hence, our study aims to analyze time trends in EO-CRC incidence rates across various US regions and to assess these trends by sex and histopathological subtypes (ADC and NET). Methods: We analyze data spanning from 2001 to 2020 from the United States Cancer Statistics (USCS) database, covering nearly 98% of the US population. Using SEER*Stat software version (8.4.2, NCI), we calculated EO-CRC incidence rates among adults aged 20–54 years, adjusting for the age standard 2000 US population. The rates were categorized by sex and US geographical regions into west, midwest, northeast, and south. Time trends, reported as annual percentage change (APC) and average APC (AAPC), were generated via Joinpoint Regression software (v.5.0.2, NCI) utilizing the weighted Bayesian Information Criteria “BIC” method to generate the best-fit trends with a two-sided *p*-value cutoff at 0.05. The rates were also stratified by histopathology into ADC and NET. Results: Between 2001 and 2020, a total of 514,875 individuals were diagnosed with early-onset CRC in the US, with 54.78% being men. Incidence rates and trends varied across geographical regions. In the western region (comprising 106,685 patients, 54.85% men), incidence rates significantly increased in both women (AAPC = 1.37, *p* < 0.001) and men (AAPC = 1.34, *p* < 0.001). Similarly, in the midwestern region (with 110,380 patients, 55.46% men), there were significant increases in incidence rates among women (AAPC = 1.06, *p* < 0.001) and men (AAPC = 1.35, *p* < 0.001). The northeastern region (with 94,758 patients, 54.53% men) also witnessed significant increases in incidence rates for both women (AAPC = 0.71, *p* < 0.001) and men (AAPC = 0.84, *p* < 0.001). In contrast, the southern region (with 203,052 patients, 54.48% men) experienced slower increases in incidence rates among both women and men (AAPC = 0.25, *p* < 0.05 in women; AAPC = 0.66, *p* < 0.05 in men). When stratified by histopathology, incidence rates for adenocarcinomas (ADC) increased in all regions, most notably in the west (AAPC = 1.45, *p* < 0.05), and least in the south (AAPC = 0.46, *p* < 0.05). Conversely, for neuroendocrine tumors (NET), while incidence rates increased similarly across all regions, the pace was notably faster compared to ADC, particularly in the west (AAPC = 3.26, *p* < 0.05) and slower in the south (AAPC = 2.24, *p* < 0.05) Discussion: Our analysis of nationwide US data spanning two decades and encompassing over half a million early-onset CRC patients, representing nearly 98% of the US population, highlights significant temporal variation in incidence rates across various geographical regions. The most substantial increases in incidence rates were observed in the west, while the least pronounced changes were noted in the south, affecting both men and women. These trends persisted across the main CRC histopathological subtypes, with NET exhibiting a notably swifter pace of increase compared with ADC. These findings hold important implications for public health strategies and underscore the need for targeted interventions to address the rising burden of early-onset CRC across different regions in the US.

## 1. Background

Colorectal cancer (CRC) is the third most common cancer in prevalence and the second leading cause of cancer-related mortality in the US [1]. While its incidence is rising worldwide, it is declining in the US [2,3,4]. CRC varies geographically, with a higher incidence in countries with a high Human Development Index (HDI) and rising rates in less-developed nations due to Western lifestyle influences [5,6]. Hungary, Slovakia, Norway, the Netherlands, and Denmark have the highest age-standardized incidence rates of CRC, whereas Guinea, Gambia, Bangladesh, Bhutan, and Burkina Faso have the lowest [5,6,7]. In the US, the northeast reports the highest incidence due to various risk factors contributing to cancer risk [8]. This has been attributed to numerous risk factors including variations in diabetes, obesity, physical inactivity, smoking, alcohol consumption, and even racial disparities, though the data on this are inconsistent [9,10,11,12].

CRC is common in older men; however, recent reports suggest a shift in the demographic of the disease with recent data revealing disease increment in the incidence of early-onset CRC (EO-CRC) among women [13,14]. This has prompted the United States Preventive Services Task Force (USPSTF) to update their guidelines to include those between the ages 45 and 49 years as candidates for CRC screening in 2021 [15].

Around 10% of CRC cases are EO-CRC with half of these cases being sporadic [16,17]. There has been increasing evidence to support the notion that EO-CRC is a different entity from late-onset CRC with respect to its clinical course [18,19,20]. While the most common histopathological subtype of EO-CRC is adenocarcinoma (ADC), recent data suggest histological variations, particularly a rise in the incidence of early-onset colorectal neuroendocrine tumors (NETs), especially in younger adults [21,22].

Similar to CRC, EO-CRC shows international variability, with South Korea experiencing the steepest rise and some countries reporting decreases in incidence such as Austria, Italy, and Lithuania. Interestingly, in Austria, CRC screening is initiated earlier at the age of 40 years [23,24]. With that in mind, regional trends in the US remain less studied, which could impact screening protocols and healthcare policies. Thus, this study aims to analyze EO-CRC incidence trends across different US regions, considering sex and histopathological subtypes.

## 2. Methods

The study provides a time trend analysis of the age-adjusted incidence rates of early-onset CRC across different geographic regions in the US between 2001 and 2020. All data utilized in our study were de-identified and publicly available, and thus, based on the National Human Research Protections Advisory Committee policy, our study was exempted from institutional review board (IRB) review.

For the purposes of this study, early-onset CRC incidence rates between 1 January 2001 and 31 December 2020 were collected from the United States Cancer Statistics (USCS) database, which is a comprehensive database contains nationwide US incidence data on cancer and approximately covers 98% of the US population [25]. Data included in the USCS database are combined from the National Program of Cancer Registries (NPCR) database and the Surveillance, Epidemiology, and End Results (SEER) database. Both the NPCR and SEER programs are fed data from central cancer registries in the US. When combined together, these two databases compose the USCS database which covers all 50 US states, the District of Columbia, and Puerto Rico [25]. To maintain high-quality and valid data, the US central cancer registries systemically export their standardized data via validated software programs and maintain the data coding per the standards of the North American Association of Central Cancer Registries [26].

Incidence rate of early-onset CRC per 100,000 population among adults aged 20–54 was chosen for our analysis, similar to previous studies [14,21,27]. The change in incidence rates over time was reported as annual percentage change (APC), which is the change in rates between two years, and the average APC (AAPC), which is as the average change in rates between 2001 and 2020. CRC histopathological subtypes in the USCS database were defined according to the International Classification of Diseases for Oncology, Third Edition Site Recode ICD-O-3/WHO 2008 as ADC with the following codes: 8140, 8141, 8143, 8144, 8210, 8211, 8213, 8220, 8221, 8255, 8260–8263, 8310, 8323, 8440, 8460, 8470, 8472, 8480–8482, 8570, 8574, and 8576; and as NET with the following codes: 8013, 8240, 8241, 8243, 8244, 8245, 8246, and 8249, as performed in prior studies [14,21]. CRC tumor location variable in the USCS database was defined according to the “Primary Site” variable in the database using the following codes: C18.0 (Cecum), C18.2 (Ascending colon), C18.3 (Hepatic Flexure of Colon), C18.4 (Transverse Colon), C18.5 (Splenic Flexure of Colon), C18.6 (Descending Colon), C18.7 (Sigmoid Colon), C18.8 (Overlapping Lesion of Colon), C18.9 (Colon Non-specified), C19.9 (Rectosigmoid Junction), and C20.9 (Rectum Non-specified), with only malignant behavior tumors included. US geographical regions were divided as in the USCS database into western, midwestern, northeastern, and southern regions.

Age-adjusted early-onset CRC incidence rates were calculated utilizing SEER*Stat software (v.8.4.2, National Cancer Institute “NCI”). The APC and AAPC were estimated using Joinpoint Regression Software (v.5.0.2, NCI) with year of diagnosis as the independent variable and incidence rates as the dependent variable. This analysis was performed via the weighted Bayesian information criteria “BIC” method, which is a statistical method used to generate time trends and is preferred due to its known flexibility and best performance [28,29,30]. A two-sided *p*-value at 0.05 was chosen as a cutoff for statistical significance. The analysis was performed after categorizing the population by sex and also after categorizing the tumors by histopathological subtype (ADC and NET).

## 3. Results

### 3.1. EO-CRC Incidence Rates and Time Trends Categorized by Sex across Different US Geographical Regions

**Figure 1:**
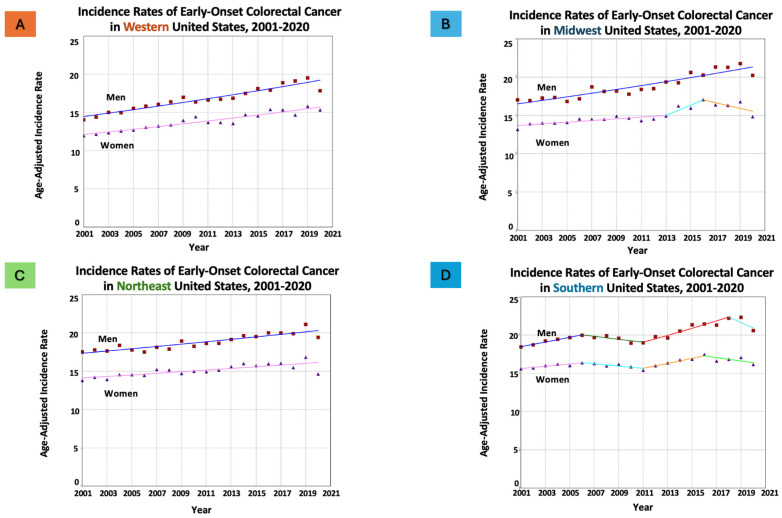
Time trends and age-adjusted incidence rates per 100,000 population for early-onset colorectal cancer (CRC) in adults aged 20–54 years categorized by US geographical regions. Different colors represent different annual percentage changes (APC).

Between 2001 and 2020, 514,875 individuals aged 20–54 years were diagnosed with early-onset CRC in the US, with 54.78% being men. When stratified by geographical regions, age-adjusted incidence rates and time trends varied (Table 1 and Figure 1). The higher incidence rates of EO-CRC were seen in the south and the lowest rates were seen in the west.

In the western region (106,685 patients; 54.85% men), early-onset CRC incidence rates per 100,000 population were significantly increasing both in women from 11.95 to 15.32 (AAPC = 1.37, *p* < 0.001) and in men from 14.02 to 17.83 (AAPC = 1.34, *p* < 0.001), respectively, over the study period.

Similarly, the midwestern region (110,380 patients; 55.46% men), also demonstrated rising incidence rates of early-onset CRC per 100,000 population in women from 13.17 to 14.83 (AAPC = 1.06, *p* < 0.001) and in men from 17.02 to 20.21 (AAPC = 1.35, *p* < 0.001), respectively, over the study period.

In the northeastern region (94,758 patients; 54.53% men), similar results were also seen with significantly increasing incidence rates of early-onset CRC in women from 13.77 to 14.62 (AAPC = 0.71, *p* < 0.001) and in men from 17.49 to 19.40 (AAPC = 0.84, *p* < 0.001).

Lastly, in the southern region (203,052 patients; 54.48% men), both genders experienced increasing incidence rate of early-onset CRC, albeit at a slower pace compared to other regions, with rates changing from 15.58 to 16.13 (AAPC = 0.25, *p* < 0.05) in women, and from 18.43 to 22.57 in men (AAPC = 0.66, *p* < 0.05) over the same study period.

### 3.2. EO-CRC Incidence Rates and Time Trends Categorized by Histopathology across Different US Geographical Regions

**Figure 2:**
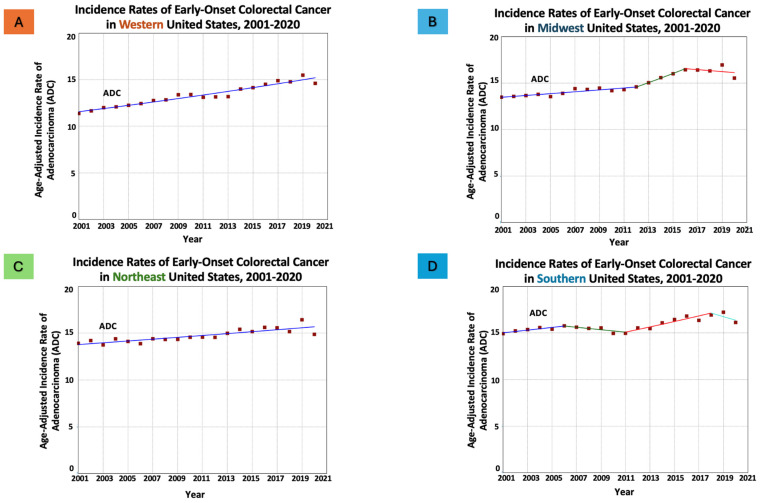
Time trends and age-adjusted incidence rates per 100,000 population for early-onset colorectal adenocarcinoma (ADC) in adults aged 20–54 years categorized by US geographical regions. Different colors represent different annual percentage changes (APC).

When stratified by histopathology, EO-CRC incidence rates experienced an upward trend in all geographical regions, albeit at varying rates (Table 2). For ADC, incidence rates per 100,000 population were increasing in all regions, most notable in the west (from 11.38 in 2001 to 14.61 in 2020; AAPC = 1.45, *p* < 0.05), and the lowest increase in the south (from 14.91 in 2001 to 16.11 in 2020; AAPC = 0.46, *p* < 0.05) (Figure 2).

**Figure 3:**
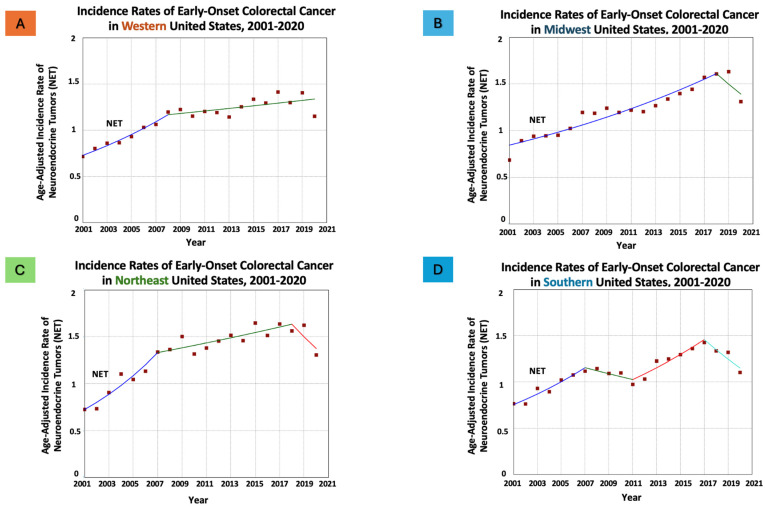
Time trends and age-adjusted incidence rates per 100,000 population for early-onset colorectal neuroendocrine tumors (NET) in adults aged 20–54 years categorized by US geographical regions. Different colors represent different annual percentage changes (APC).

Conversely, for NET, while similar increasing trends were observed in all regions, the pace was higher compared with ADC. Particularly, the west and northeast exhibited the highest increase (west: from 0.71 in 2001 to 1.15 in 2020, AAPC = 3.26, *p* < 0.05; and northeast: from 0.73 in 2001 to 1.30 in 2020, AAPC = 3.43, *p* < 0.05), while the south showed the least increase (from 0.77 in 2001 to 1.10 in 2020, AAPC = 2.24, *p* < 0.05) (Figure 3).

## 4. Discussion

**Figure 4:**
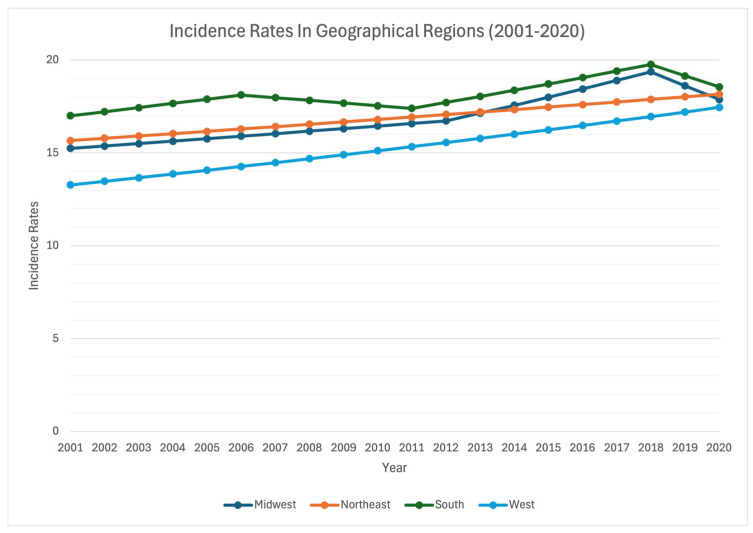
Time Trend and Age-Adjusted Incidence Rates per 100,000 Population for Early-Onset Colorectal Cancer (EO-CRC) in Adults Aged 20–54 years Categorized by US Geographical Region.

Our nationwide analysis reveals a consistent increase in age-adjusted incidence rates of EO-CRC among both genders and across all geographical regions in the US, encompassing the west, midwest, northeast, and south. The most substantial rise occurred in the western region, with a comparatively smaller increase in the southern region (Figure 4). When categorized by histopathological subtype, both ADC and NET types displayed upward trends across all regions, with NET exhibiting a more pronounced acceleration in increase compared with ADC, especially in the west and northeast, and least notable in the south of the US.

The interplay of geographic location to colorectal cancer risk is likely multifaceted. Previous studies proposed that local factors such as obesity, smoking, red meat consumption, and diabetes could contribute to observed disparities in CRC incidence and survival rates. The last SEER-based analysis of EOCRC incidence rates incorporating geographical differences was conducted by Abualkhair et al. between 2000 and 2015 [31]. Notably, this study has highlighted that the southern United States has the highest incidence of EO-CRC, which is consistent with our study’s observation. This finding also aligns with the presence of various risk factors in the southern regions, including high levels of physical inactivity, poverty, and greater disease burden such as chronic kidney and cardiovascular diseases [9]. Other factors that may contribute to the higher incidence of EO-CRC in the southern US may include the higher rate of uninsured individuals in the south compared to other regions [32]. This may prevent patients in the south from presenting to clinics and addressing any potential CRC risk factors such as tobacco use disorder, alcohol use disorder, obesity, or even any colorectal polyps. Moreover, prior data showed that rural south areas tend to have the lowest rates of physicians compared to rural areas in other geographical regions in the US [33]. All of these factors have led to higher rates of morbidity and mortality in the south. Our findings represent the first to highlight that while the southern regions continue to harbor the highest number of EO-CRC cases, the rate of increase in incidence is comparatively slower than other geographic regions.

Similarly, our data findings indicate that a significant steep rise in EO-CRC incidence rates in the western, midwestern, and northeastern US may suggest overlooked risk factors specific to these areas. Historically, Primary prevention of CRC has largely focused on addressing modifiable risk factors such as obesity, smoking, and alcohol consumption. A previous study investigating EO-CRC mortality have identified the existence of CRC “hot spots” concentrated in the south [34]. The authors identified 232 hotspots for EO-CRC in the US using CDC mortality data, out of which there were 14 of them (92%) in the south [34]. Furthermore, prior nationwide data showed that the rates of cigarette smoking is the highest in the south and the midwest [34]. Our current data shed light on the rising incidence of EO-CRC in different geographical regions and imply a need for increased attention to vulnerable populations in different geographic regions to ensure comprehensive early interventions with the hope of preventing EO-CRC and improving outcomes.

Notably, there is evidence suggestive of a link between reduced sunlight exposure (i.e., associated with higher geographic latitudes) and lower levels of serum vitamin D and calcium, and an increased risk of CRC [35]. This observation raises the possibility of an overlooked risk factor in the northeast, where states at higher latitudes and with greater metropolitan/industrial density might experience decreased sun exposure. However, this does not entirely account for the rapid rise of EO-CRC incidence observed in the west, given its diverse geographical positioning. Consequently, further research is warranted to understand the impact of sunlight exposure from various regions in the US and the incidence of early-onset colorectal cancer (EO-CRC).

Additionally, other modifiable risk factors fail to fully elucidate the sharper rise of EO-CRC incidence observed in the west. For instance, antibiotic exposure, is theorized to impact the gut microbiome which can lead to a “Dysbiosis state” promoting cancer formation [36,37]. On the contrary, CDC data for outpatient antibiotic prescriptions from 2011 to 2022 demonstrates the lowest prescription rates in western states and the highest rates in the south [38].

Another proposed explanation for the general increase in EO-CRC incidence is increased detection due to more gastrointestinal procedures performed such as colonoscopies and sigmoidoscopies, especially in younger adults. In 2017, Murphy et al. demonstrated that the rise of colonoscopy rates in younger adults between 2001 and 2014 correlated with the rise in early-onset incidence of CRC [39]. Nonetheless, despite evidence correlating rising gastrointestinal procedure rates with EO-CRC incidence, the western US demonstrates the lowest rates of these procedures compared to other regions.

Based on our study and the existing literature, pinpointing a definitive explanation for the accelerated rise of EO-CRC in the west remains challenging. Investigating the potential contributing factors in this region presents a substantial area of future research, as no predominant risk factors specific to the western US have been identified to date.

Furthermore, there is a scarcity of data on geographic trends of early-onset CRC based on histopathology (ADC vs. NET). A large cross-sectional study of 19,669 patients with gastrointestinal NETs between 1975 and 2008 revealed increasing incidence rates across all geographic regions in the US [40]. More specifically, rates of distant appendiceal NET were noted to be higher in the west, while rates of distant colonic NETs were higher in the south. Our study emphasizes and builds on these findings by examining more recent trends of colorectal ADC and colorectal NET incidence, using a larger sample size. Investigating different histopathological subtypes of EO-CRC is essential given the variation in risk factors, progression, and outcomes of ADC and NET.

Several strengths characterize our study. We leveraged the most comprehensive US cancer incidence database, analyzing over half a million cases of early-onset CRC diagnosed between 2001 and 2020. Furthermore, our study provides insights into the interplay of sex, histopathology, and geographical regions in EO-CRC. Employing joinpoint regression for time trend analysis via the modified Bayesian information criteria (BIC) methods, we utilized statistical techniques recommended for nationwide databases, known for their flexibility across different analysis scenarios and ability to detect trend changes [29,41]. Some of the limitations of our study include the absence of clinical variables associated with the risk of early-onset CRC. However, our analysis is observational and aims to delineate recent epidemiological trends, guiding future research efforts toward further investigations of this topic and geographical variations. Moreover, limitations inherent to large databases such as the possibility of miscoding and record loss, may affect our findings [42]. Nevertheless, the USCS database utilized in our study undergoes a rigorous analysis process to ensure data validity and minimize errors before publication [25].

Our nationwide US data analysis covering nearly 98% of the US population, reveals a temporal variation in the incidence changes in early-onset colorectal cancer across different geographical regions. We demonstrate increasing incidence rates of early-onset CRC among men and women in the western, midwestern, northeastern, and southern regions, with the most significant rises occurring in the west and the slowest in the south. While the south exhibited the highest incidence of EO-CRC, rates increased at a slower pace compared to other geographical regions. These findings both persisted across both main CRC histopathological subtypes (ADC and NET), with the NETs showing a more pronounced increase in rates compared to ADC. Our findings hold public health implications, prompting further research to explore and investigate any potential disproportional exposure to region-specific risk factors over the past two decades, particularly in the western region of the US.

## Figures and Tables

**Table 1:** Changes in time trends of early-onset colorectal cancer (CRC) incidence rates in the US between 2001 and 2020 in different geographical regions.

Sex	Early-Onset CRC Cases (N = 514,875) ^a^	Trends ^b^		
		Time Period	APC (95% CI)	AAPC (95% CI)
**West**				
Women	48,166 (9.35%)	2001–2020	1.37 * (1.12 to 1.64)	1.37 * (1.12 to 1.64)
Men	58,519 (11.37%)	2001–2020	1.50 * (1.23 to 1.79)	1.50 * (1.23 to 1.79)
**Midwest**				
Women	49,159 (9.55%)	2001–2013	0.77 (−0.92 to 1.19)	0.68 * (0.26 to 0.92)
		2013–2016	4.33 * (1.57 to 5.88)	
		2016–2020	−2.26 * (−6.13 to −0.62)	
Men	61,221 (11.89%)	2001–2020	1.35 * (0.96 to 1.76)	1.35 * (0.96 to 1.76)
**Northeast**				
Women	43,088 (8.37%)	2001–2020	0.71 * (0.40 to 1.01)	0.71 * (0.40 to 1.01)
Men	51,670 (10.04%)	2001–2020	0.84 * (0.60 to 1.08)	0.84 * (0.60 to 1.08)
**South**				
Women	92,427 (17.95%)	2001–2006	0.97 * (0.37 to 2.56)	0.25 * (0.09 to 0.43)
		2006–2011	−0.91 * (−2.32 to −0.24)	
		2011–2016	2.01 * (1.25 to 3.54)	
		2016–2020	−1.33 * (−2.81 to −0.45)	
Men	110,625 (21.49%)	2001–2006	1.62 * (0.84 to 3.52)	0.66 * (0.45 to 0.89)
		2006–2011	−0.98 * (−2.67 to −0.07)	
		2011–2018	2.31 * (1.84 to 3.64)	
		2018–2020	−3.26 * (−5.32 to −0.55)	

^a^ Data are presented as count numbers followed by percentages of the count numbers from the total cases of early-onset CRC in the database. ^b^ Time trends were computed using Joinpoint Regression Program (v5.0.2, NCI) with three maximum joinpoints allowed (four-line segments). * Implies statistical significance (*p*-value < 0.05).

**Table 2:** Changes in Time trends of early-onset colorectal cancer (CRC) incidence rates in the US between 2001 and 2020 in different geographical regions stratified by histopathology into adenocarcinoma (ADC) and neuroendocrine tumors (NET).

Sex	Early-Onset CRC Cases (N = 514,875) ^a^	Trends ^b^		
		Time Period	APC (95% CI)	AAPC (95% CI)
**West**				
ADC	92,818 (18.03%)	2001–2020	1.45 * (1.27 to 1.64)	1.45 * (1.27 to 1.64)
NET	8036 (1.56%)	2001–2008	6.99 * (4.77 to 11.78)	3.26 * (2.60 to 4.14)
		2008–2020	1.14 (−0.22 to 2.03)	
**Midwest**				
ADC	96,814 (18.80%)	2001–2012	0.72 * (0.24 to 1.00)	0.95 * (0.74 to 1.12)
		2012–2016	3.23 * (1.98 to 4.77)	
		2016–2020	−0.65 (−3.12 to 0.45)	
NET	8040 (1.56%)	2001–2018	3.87 * (3.24 to 7.83)	2.66 * (1.82 to 4.06)
		2018–2020	−7.06 (−14.69 to 3.14)	
**Northeast**				
ADC	82,461 (16.02%)	2001–2020	0.68 * (0.45 to 0.91)	0.68 * (0.45 to 0.91)
NET	7463 (1.45%)	2001–2007	10.67 * (1.03 to 21.76)	3.43 * (2.29 to 4.97)
		2007–2018	1.89 (−0.46 to 18.81)	
		2018–2020	−8.32 (−18.18 to 1.98)	
**South**				
ADC	176,577 (34.30%)	2001–2006	0.96 (−0.02 to 3.72)	0.46 * (0.18 to 0.76)
		2006–2011	−0.85 (−2.81 to 0.41)	
		2011–2018	1.84 * (1.30 to 4.14)	
		2018–2020	−2.28 (−5.17 to 0.48)	
NET	12,666 (2.46%)	2001–2007	7.32 * (5.32 to 11.83)	2.24 * (1.49 to 3.06)
		2007–2011	−2.90 (−7.43 to 0.69)	
		2011–2017	6.00 * (4.18 to 12.25)	
		2017–2020	−7.54 * (−14.00 to −3.14)	

^a^ Data are presented as count numbers followed by percentages of the count numbers from the total cases of early-onset CRC in the database. ^b^ Time trends were computed using Joinpoint Regression Program (v5.0.2, NCI) with three maximum joinpoints allowed (four-line segments). * Implies statistical significance (*p* < 0.05).

## Data Availability

Data included in this work can be obtained from the United States Cancer Statistics (USCS) database, which is a publicly available database.
